# Valve-sparing aortic root replacement after closure of ventricular septal defect

**DOI:** 10.1016/j.xjtc.2024.08.026

**Published:** 2024-09-06

**Authors:** Atsunori Kono, Yoshikatsu Nomura, Hirohisa Murakami, Toshihito Sakamoto, Hiroshi Tanaka

**Affiliations:** Department of Cardiovascular Surgery, Hyogo Prefectural Harima Himeji General Medical Center, Hyogo, Japan

**Figure:**
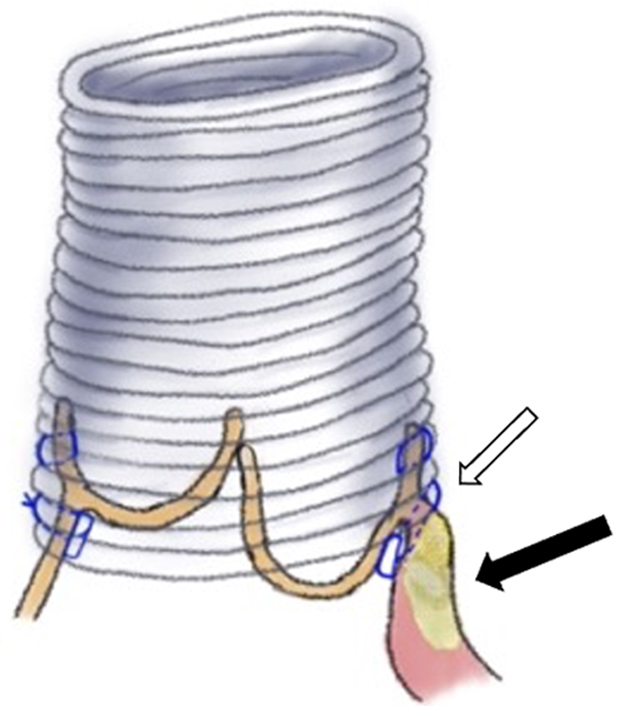
Higher proximal sutures due to calcification cause prolapse of the right coronary cusp.

**Central Message**

It is sometimes challenging to dissect the right ventricular wall deep enough in patients with a history of closure of VSD during reimplantation, which causes cusp prolapse.

Valve-sparing aortic root replacement in patients with congenital heart disease has been reported, describing the technical difficulties of the procedure.^1^ Three adult patients with a history of closure of ventricular septal defect (VSD) underwent valve-sparing root replacement using the reimplantation technique for the root aneurysm with aortic regurgitation (AR). The institutional review board approved this study and waived written informed consent (No. R6-60; August 1, 2024).

## Case 1

**Figure 1:**
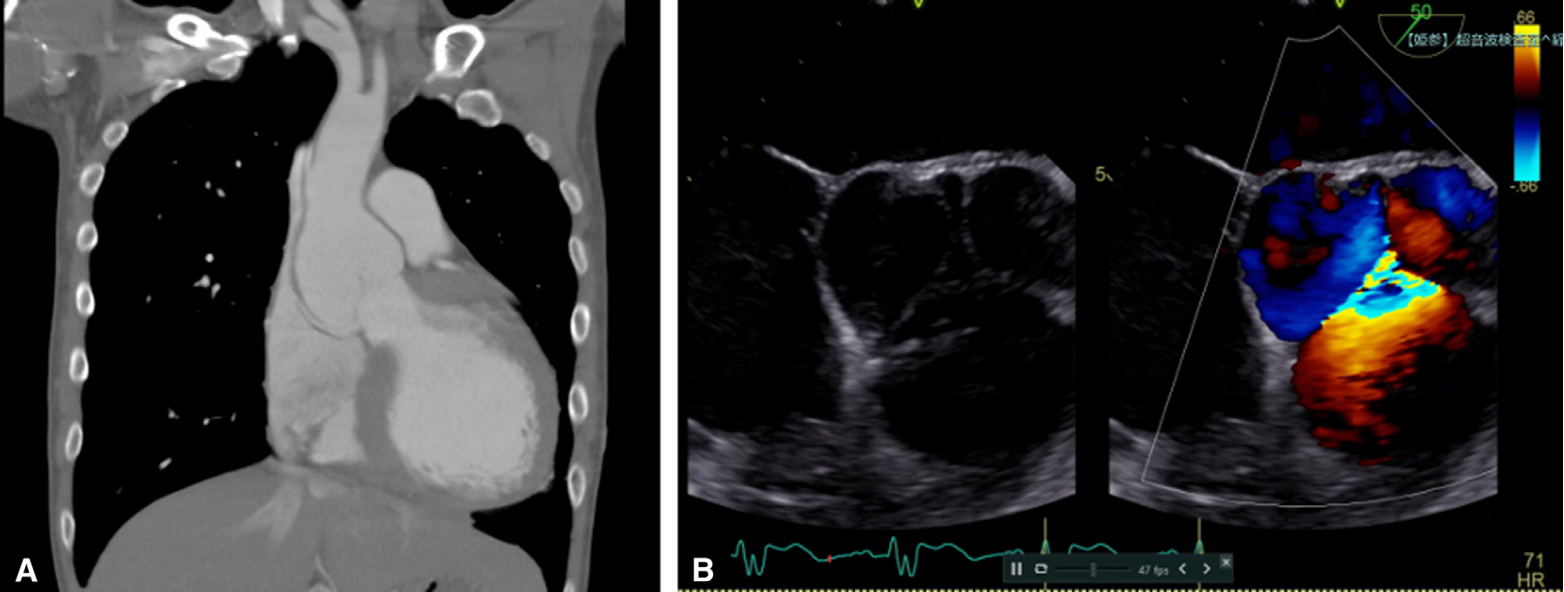
A, Preoperative computed tomography showed a root aneurysm. B, Preoperative transesophageal echocardiography showed severe aortic regurgitation and prolapse of the right coronary cusp with bending.

**Figure 2:**
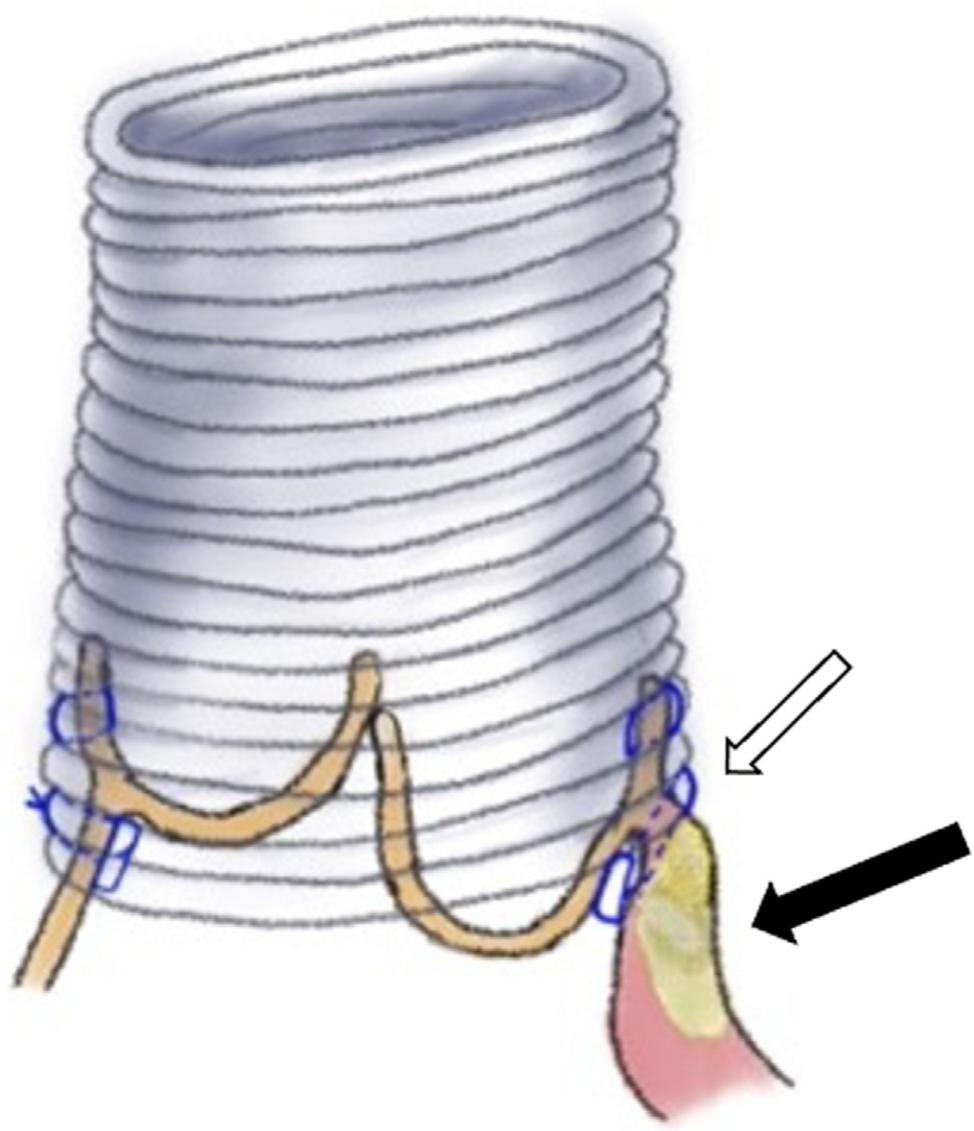
If the dissection of the right ventricular wall from the interventricular septum is not deep enough to place the proximal sutures below the annulus due to the calcification, the graft lies above the annulus (*white arrow*), and the right coronary cusp shows billowing or prolapsing toward the left ventricular outflow. The *black arrow* shows calcification.

**Figure E1:**
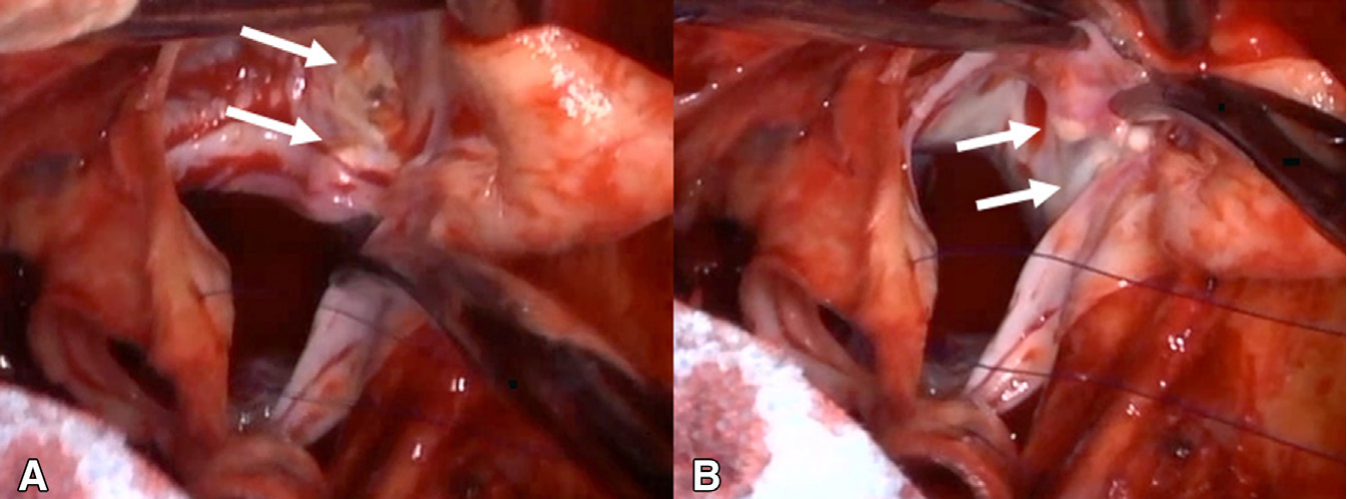
Intraoperative findings. A, there was calcification around the right-noncoronary cusp commissure (*arrows*). B, calcification below the subcommissural triangle of the right-noncoronary cusps (*arrows*).

**Figure E2:**
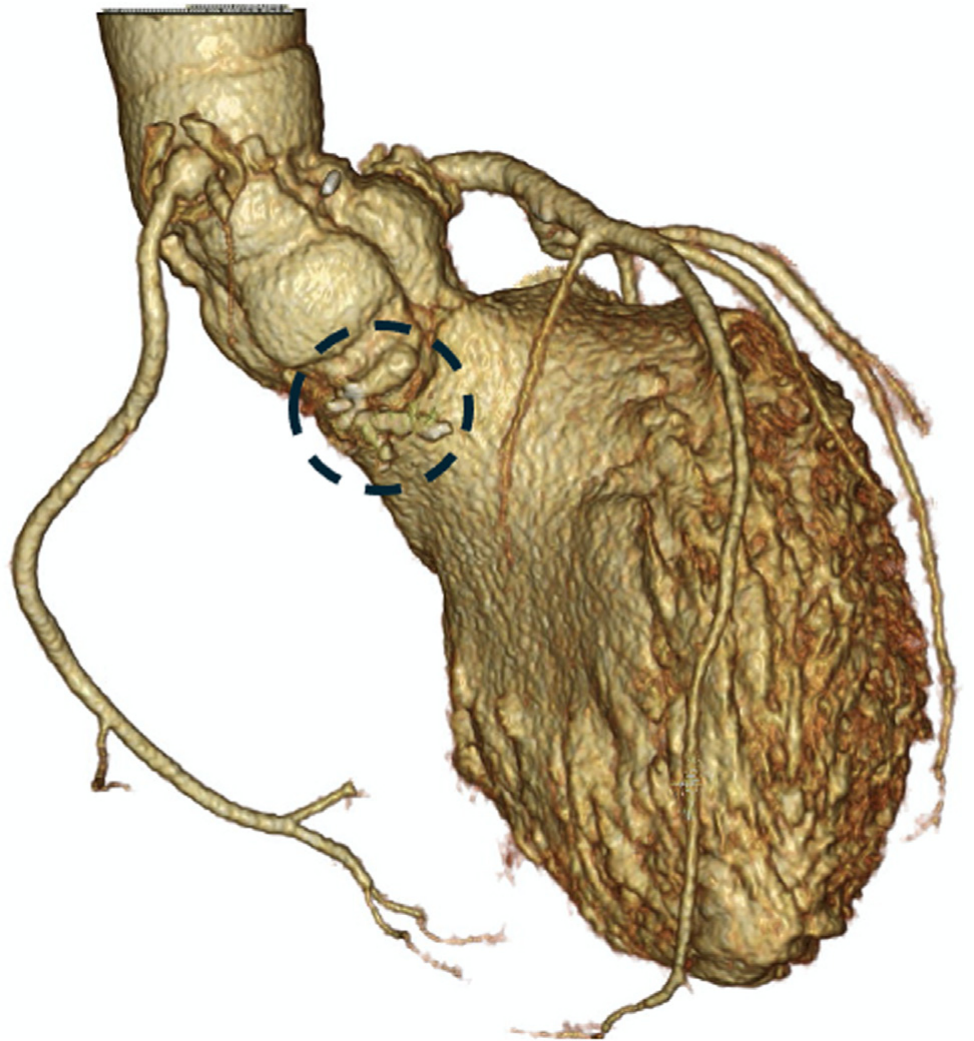
Postoperative computed tomography showed the calcification in the left ventricular outflow below the *bottom* of the graft (*dotted circle*).

A 42-year-old man with a history of the closure of VSD and patent ductus arteriosus at age 6 months presented with congestive heart failure. Echocardiography showed reduced ejection fraction of 40%, severe AR, and aortic root aneurysm (Figure 1, *A* and *B*). He underwent valve-sparing root replacement using the reimplantation technique. Intraoperatively, there was calcification around the right commissure and prolapse of the right coronary cusp with cusp bending (Figure E1, *A* and *B*). It was impossible to dissect the right ventricular wall from the interventricular septum down to the level of the nadir of the right coronary cusp because of the calcification, and the proximal sutures were placed higher than usual (Figure 2). Correction of the right coronary cusp prolapse could not be achieved with cusp plication alone but by adding free margin resuspension. Postoperative echocardiography showed trivial AR, and computed tomography showed calcification below the bottom of the graft (Figure E2).

## Case 2

**Figure E3:**
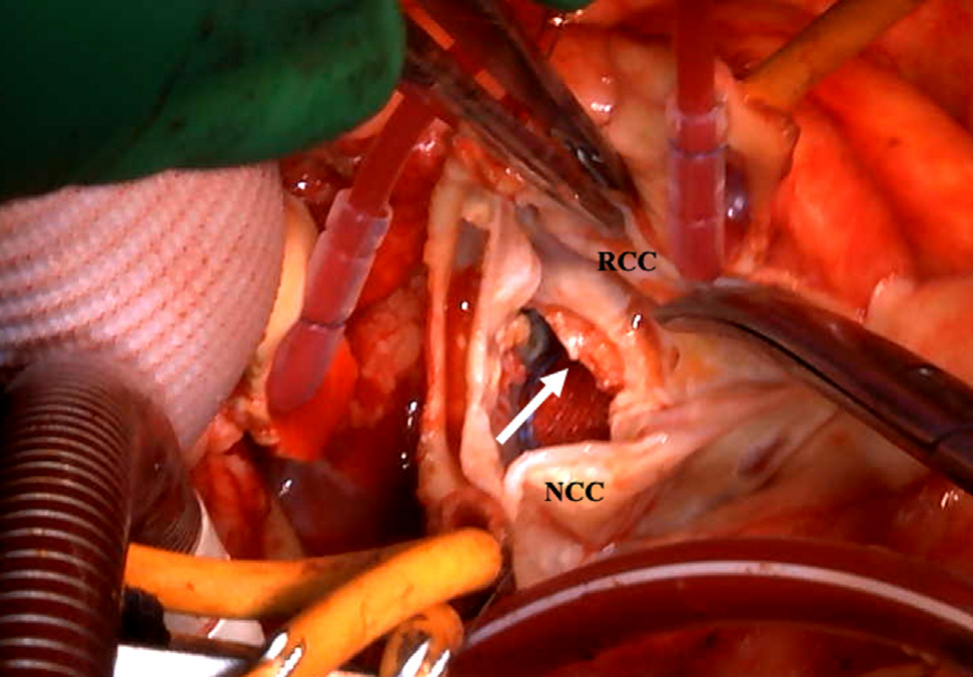
Intraoperative findings showed there was a severely calcified band in the left ventricular outflow tract (subaortic stenosis) (*white arrow*) and involvement of the noncoronary cusp (*NCC*) to the calcified band, which would cause aortic regurgitation. *RCC*, Right coronary cusp.

A 52-year-old man with a history of the closure of perimembranous VSD at age 3 years was referred to our hospital for severe AR and moderate aortic stenosis with aortic root aneurysm. He underwent the valve-sparing root replacement using the reimplantation technique. Intraoperatively, a severely calcified band in the left ventricular outflow tract (subaortic stenosis) and involvement of the noncoronary cusp to the band caused aortic regurgitation (Figure E3). Dissecting the right ventricular wall from the interventricular septum to the nadir level of the right coronary cusp could not be achieved due to calcification. We resected the calcified band and released the involvement of the noncoronary cusp, and the reimplantation was performed with a higher proximal suture line in the right coronary sinus. The prolapse of the right coronary cusp was noted after completion of the reimplantation, and free margin resuspension was performed to correct the prolapse. Postoperative echocardiography showed trivial AR.

## Case 3

**Figure E4:**
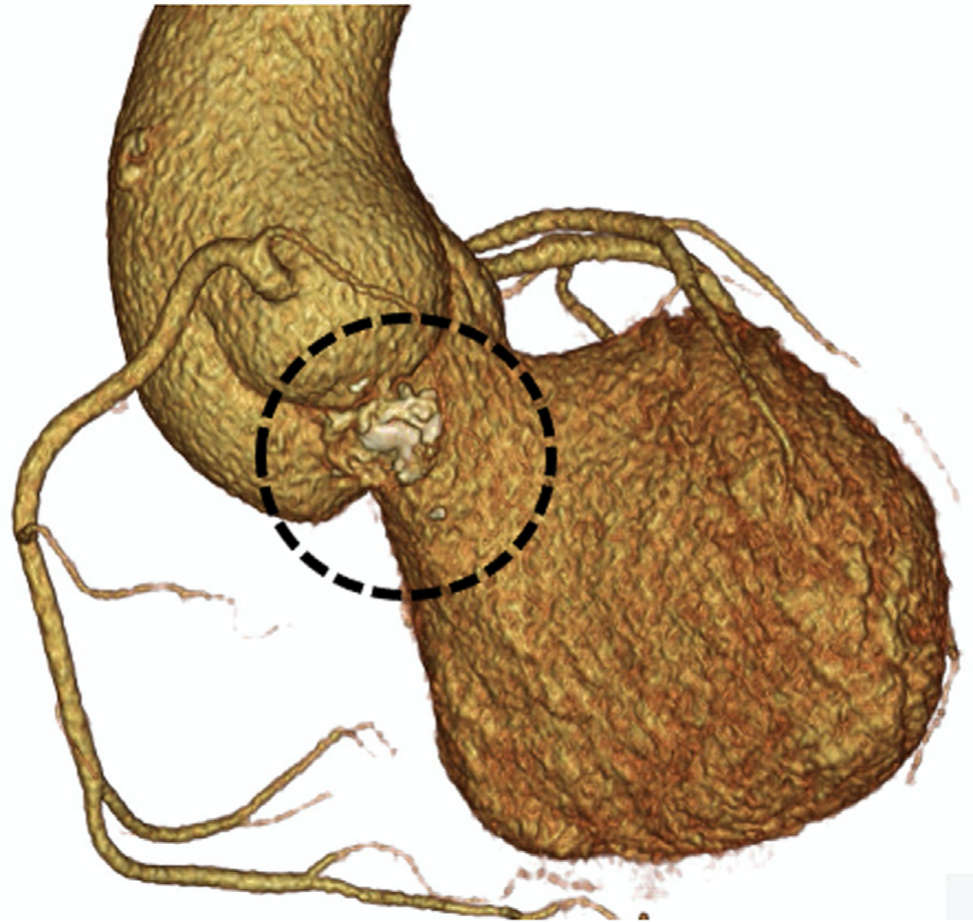
Preoperative computed tomography showed the calcification in the left ventricular outflow below the subcommisural triangle of the right coronary cusp (*dotted circle*).

A 47-year-old man with a history of closure of perimembranous VSD at age 6 months was presented with a complaint of dyspnea. The electrocardiogram showed a complete atrioventricular block with a heart rate of 40/minute, and echocardiography showed aortic root aneurysm and moderate AR. Computed tomography showed a root aneurysm and calcification in the left ventricular outflow (Figure E4). He underwent aortic root reimplantation and pacemaker implantation. Similar to the previous 2 patients, the proximal sutures were placed higher than usual. He required free margin resuspension as a cusp repair, and postoperative echocardiography showed no AR.

## Comments

In the reimplantation technique, deep dissection of the right ventricular wall from the interventricular septum and placing the proximal sutures below the basal ring are essential parts of the procedure. If this is not implemented adequately, the graft lies at the same level as the basal ring or above it, which causes the right coronary cusp prolapse.^2^ These 3 patients underwent the closure of the perimembranous VSD using mattress sutures with polytetrafluoroethylene pledgets. The materials caused calcification and adhesion of the right ventricular muscle; therefore, we were forced to place the proximal sutures higher than usual below the right coronary cusp. Consequently, the right coronary cusp was billowing to the left ventricular outflow and prolapsing (Figure 2). In this scenario, the cusp is not stretched, and there is no redundant free margin length. Correcting the prolapse by using cusp plication alone is difficult. We used free margin resuspension by polytetrafluoroethylene suture (Gore-Tex; W.L. Gore and Associates) and obtained good results. The cusp plication technique is widely used to correct the prolapsing cusps, and most of them are stretched and have redundant free margin length, which has room to plicate. A few articles reported that free margin resuspension (reinforcement) is more promising than central plication.^3^^,^^4^ The technique is complicated but has the advantage of avoiding overcorrecting the free margin length to obtain sufficient effective height.

In summary, it is sometimes challenging to dissect the right ventricular wall deep enough from the interventricular septum in patients with a history of closure of VSD in the reimplantation. A higher proximal suture line results in prolapse of the right coronary cusp, but in that case, free margin resuspension would be effective.

## Conflict of Interest Statement

The authors reported no conflicts of interest.

The *Journal* policy requires editors and reviewers to disclose conflicts of interest and to decline handling or reviewing manuscripts for which they may have a conflict of interest. The editors and reviewers of this article have no conflicts of interest.
